# Yellow Fluorescent Protein-Based Assay to Measure GABA_A_ Channel Activation and Allosteric Modulation in CHO-K1 Cells

**DOI:** 10.1371/journal.pone.0059429

**Published:** 2013-03-14

**Authors:** Teres Johansson, Tyrrell Norris, Helena Peilot-Sjögren

**Affiliations:** AstraZeneca Research & Development, Discovery Sciences, Cellular Reagents & Assay Development, Mölndal, Sweden; McLean Hospital/Harvard Medical School, United States of America

## Abstract

The γ-aminobutyric acid A (GABA_A_) ion channels are important drug targets for treatment of neurological and psychiatric disorders. Finding GABA_A_ channel subtype selective allosteric modulators could lead to new improved treatments. However, the progress in this area has been obstructed by the challenging task of developing functional assays to support screening efforts and the generation of cells expressing functional GABA_A_ ion channels with the desired subtype composition. To address these challenges, we developed a yellow fluorescent protein (YFP)-based assay to be able to study allosteric modulation of the GABA_A_ ion channel using cryopreserved, transiently transfected, assay-ready cells. We show for the first time how the MaxCyte STX electroporation instrument can be used to generate CHO-K1 cells expressing functional GABA_A_ α2β3γ2 along with a halide sensing YFP-H148Q/I152L (YFP-GABA_A2_ cells). As a basis for a cell-based assay capable of detecting allosteric modulators, experiments with antagonist, ion channel blocker and modulators were used to verify GABA_A_ subunit composition and functionality. We found that the I^−^ concentration used in the YFP assay affected both basal quench of YFP and potency of GABA. For the first time the assay was used to study modulation of GABA with 7 known modulators where statistical analysis showed that the assay can distinguish modulatory pEC_50_ differences of 0.15. In conclusion, the YFP assay proved to be a robust, reproducible and inexpensive assay. These data provide evidence that the assay is suitable for high throughput screening (HTS) and could be used to discover novel modulators acting on GABA_A_ ion channels.

## Introduction

GABA_A_ ion channels mediate inhibitory neurotransmission in the central nervous system. They are members of the pentameric, ligand-gated ion channel family composed of around 18 different subunits which can give rise to a high variability in subunit composition, function and pharmacology [1], [2]. The most abundant form in the brain is composed of two α subunits, two β subunits and one γ subunit [3], [4], where the two most widely expressed subtypes are α1β2γ2 and α2β3γ2 [5]. In recent studies it has been demonstrated that, in general, α2/α3 containing channels mediate anxiolytic effects, α1 containing channels mediate sedative effects and α5 containing channels are involved in memory and learning [6]. The GABA agonist binding site is located at the interface between the α and β subunits and the benzodiazepine site, one of several modulator sites, is found at the interface between the α and γ subunits [1]. GABA_A_ ion channels are important drug targets for neurological and psychiatric disorders such as sedation, anaesthesia, epilepsy, anxiolysis and muscle relaxation. Nevertheless, there is scope for development of new and improved drugs with subtype selectivity to overcome unwanted side effects of current treatments [7], [8]. The aim of this study was to develop a sensitive and robust assay to be able to monitor GABA_A_ ion channel modulation and identify compounds that could address this unmet medical need.

There are several techniques for monitoring activity of Cl^−^ channels such as GABA_A_. For a functional assay to be suitable for HTS of large compound libraries it should preferably be sensitive, fast, inexpensive and adaptable to high-density format. Patch-clamp electrophysiology is considered the “gold standard” for studying ion channels due to its sensitivity, however, it is costly and often limited in throughput. Binding assays are simple and fast but do not necessarily correlate with functional activity. Membrane potential assays using fast responding fluorescent dyes show medium to high sensitivity, give a robust signal under HTS conditions, have a good dynamic range and are high throughput [9], however, they require dye-loading steps and are relatively expensive. Moreover, there is evidence that some membrane potential dyes directly modulate the GABA_A_ ion channel, [10] thereby introducing a risk of generating false positive hits in screening. Considering limitations with existing screening techniques and the difficulty in finding novel GABA_A_ modulators we established a YFP assay to study GABA_A_ activity and modulation. The method was developed by Galietta and colleagues [11], [12] where YFP was used as an intracellular sensor for monitoring anionic flux through the cystic fibrosis transmembrane conductance regulator (CFTR) protein. The assay was later adapted to HTS for CFTR modulators by Sui et al. [13]. YFP is an engineered variant of green fluorescent protein (GFP) carrying four point mutations (S65G, V68L, S72A, T203Y). Random mutation approaches have identified mutations, H148Q and I152L, that further increase the YFP halide sensitivity [12], [14]. Upon ion channel activation anions such as I^−^, NO_3_^−^, Br^−^ and Cl^−^ enter the cell, bind to YFP and quench its fluorescence. Agonist-dependent quench of YFP fluorescence can then be measured with a fluorescent reader and used to determine channel activation, inhibition and modulation. The YFP assay has the benefit of being a noninvasive technique with the ability to measure fast responses. In addition, ions flux in the natural direction of the GABA_A_ ion channel in comparison to a membrane potential assay where the electrochemical gradient is artificially polarized and a chloride efflux is measured. Furthermore, with no external addition of fluorescent dye the cost is reduced and the risk of GABA modulatory effects from the dye is avoided. A significant advantage of the YFP assay is that it does not require stable cell lines to obtain full dynamic range as only transfected cells produce a signal and simultaneous transfection of YFP-H148Q/I152L and ion channel subunits show high degree of co-expression [15]. Exogenous dyes, on the other hand, are taken up by all cells irrespective of whether they express the target ion channel, thereby reducing the dynamic range.

Difficulties in establishing stable cell lines along with the large investment in time and resources have made large scale transient transfection an attractive way of generating cells for assays. Among transfection technologies electroporation is an efficient way of introducing DNA into cells without the need for chemical transfection reagents. The mechanism for DNA uptake into the cell involves electrophoretic association of DNA with the cell membrane followed by DNA insertion in the membrane and entry into the cell [16]. Due to limitations in scaling up it has previously been difficult to transfect large enough cell batches for screening. In this study we overcome this problem by using MaxCyte STX (MaxCyte, Inc.). The instrument enables small to medium scale static electroporation of 0.5×10^7^–3.5×10^8^ cells for optimization work and large scale flow electroporation of up to 10^10^ cells with high transfection efficiency and reproducibility.

The YFP assay has previously been developed for CFTR [11], [13], Glycine [15] and rat GABA_A_ [15], [17]. The goal of our work was to develop a YFP assay for human GABA_A_ α2β3γ2 expressed transiently in Chinese Hamster Ovary (CHO-K1) cells, to confirm the applicability of cryopreserved transiently transfected cells, and to test the assay's suitability for screening of modulators. We found that cryopreserved transiently transfected assay-ready cells showed robust performance in the YFP assay and enabled easy handling and scale-up. Our results show that the YFP assay can be established to study agonist activation and modulation of the GABA_A_ channel.

## Materials and Methods

### Chemicals and Reagents

Diazepam, Alpidem, Lorazepam, Clobazam, Desmethyl-clobazam, TPA-023, L-838417 and Bicuculline were synthesized at AstraZeneca. Ham's F-12 medium, Picrotoxin, GABA and all other chemicals were purchased from Sigma.

### Cloning

The cDNA of YFP (Genbank accession number AY818378) with H148Q and I152L mutations was inserted into the pcDNA3.1 vector using 5′ BamHI and 3′ NotI sites. Human GABA_A_ subunits α2, β3 and γ2 (Genbank accession numbers; α2 NM_000807, β3 NM_000814, γ2 NM_000816) were inserted into pGen-IRES-neo2 vectors [18] using NotI 5′ and 3′ sites.

### Cell Culture and Transfection

CHO-K1 cell line (ATCC, CCL-61) was cultured in Ham's F-12 medium supplemented with 10% fetal bovine serum and incubated at 37°C and 5% CO_2_. Transient transfection was performed using MaxCyte STX electroporation instrument (MaxCyte, Inc.) with CHO cell line specific electroporation protocol. The cells were split one day prior to electroporation to ensure that cells were in the log phase growth at time of transfection. Cells were harvested with Accutase (PAA Laboratories), pelleted at 250 g for 10 minutes, rinsed with MaxCyte Electroporation Buffer (MaxCyte, Inc.), pelleted again and resuspended in MaxCyte Electroporation Buffer at a density of 1×10^8^ cells/ml. CHO-K1 cells were electroporated simultaneously with four plasmids using a total amount of 300 µg DNA per 1×10^8^ cells; YFP-H148Q/I152L (150 µg) and GABA_A_ (72.5 µg α2, 5 µg β3 and 72.5 µg γ2) plasmids. Following electroporation, cells were incubated at 37°C and 5% CO_2_ for 20 minutes to recover. Cells were then diluted in culture medium and centrifuged 250 g for 10 minutes. Pelleted cells were resuspended to 5–10×10^6^ cells/ml in Ham's F12 with 20% fetal bovine serum and 10% DMSO, and cryopreserved using controlled-rate cryo containers (Nalgene, Thermo Scientific). Cryopreserved assay-ready cells were used for all experiments.

### YFP-based Assay

Cryopreserved transiently transfected cells were thawed and plated in 384-well poly-D-lysine-coated plates at a density of 15000 cells/well using a Multidrop Combi Reagent Dispenser (Thermo Scientific). After 24 h incubation at 37°C and 5% CO_2_, cells were washed three times on an ELx405 Select Deep Well Microplate Washer (BioTEK), exchanging 50 µl culture medium with 30 µl HBSS buffer supplemented with 20 mM Hepes. Plates were incubated with buffer for 1 h at 37°C and 5% CO_2_. Cell plates were assayed on a FLIPR Tetra (Molecular Devices) using excitation filter of 470–495 nm and emission filter of 515–575 nm. Starting with 30 µl buffer in each well, cells were pre-incubated with 10 µl compound for 15 minutes. 20 µl GABA in NaI buffer pH 7.3 (140 mM NaX, 20 mM Hepes, 5 mM KCl, 2 mM CaCl_2_, 1 mM MgCl_2_ where NaX was a mix of NaCl and NaI) was added to activate the ion channel and induce an agonist-dependent quench of YFP fluorescence. Data was acquired for 120 s with fluorescence recording at 1 s intervals where GABA/NaI solution was added after 10 s of baseline reading. Compounds were initially solubilized to a concentration of 10 mM in DMSO and then diluted in HBSS buffer with 20 mM Hepes. Final DMSO concentration in the assay was 0.02% for modulators and at 1% for Picrotoxin and Bicuculline. DMSO only controls were included in every experiment and there was no detectable effect of DMSO on GABA channel activation. GABA solutions were prepared fresh for every experiment and 3-fold serial dilutions were performed on a 384-well microplate in NaI buffer using a Biomek FX (Beckman Coulter) liquid handling device.

### Data Analysis

Data at 1–10 s was adjusted to a baseline of 100% and results were then extracted as % quench at 120 s. % quench is defined as (F_init_ – F_final_)/F_init_*100 where F_init_ and F_final_ are the initial and final values of fluorescence respectively. GABA concentration-response curves were fitted with the Hill equation, F  =  F_init_/(1+(EC_50_/[GABA])^nH^), where F is the fluorescence corresponding to a GABA concentration [GABA], F_init_ is the initial fluorescence value, EC_50_ is the concentration that elicits half-maximal response and nH is the Hill coefficient. Curve fits were performed using least-squares fitting routine (GraphPad Prism 5, GraphPad Software Inc.).

### Statistical Analysis

To evaluate the statistical significance of pEC_50_ differences seen for different I^−^ concentrations, Analysis of Variance was used. This allowed for I^−^ concentration and experimental occasion, to enable t-tests, to use a pooled estimate of variability when comparing I^−^ concentrations. Two-sided tests were used.

To calculate the pEC_50_ difference at which two modulators can be considered to have statistically significant differences in GABA potency, 7 known modulators were tested in the YFP assay on 3 different occasions. A pooled estimate of variability was used to calculate the standard error of the difference between two compounds' average of 3 occasions pEC_50_. The 95% confidence interval was calculated as 2× this standard error. Taking the anti-log converts this to a fold change (ratio) between two EC_50_ values.

## Results

### Optimization of Transient Transfection

To generate cells expressing the GABA_A_ ion channel for the YFP assay, four different plasmids (GABA_A_ α2, β3, γ2 and YFP-H148Q/I152L) were electroporated simultaneously using MaxCyte STX. DNA ratio for subunits of GABA_A_ α2β3γ2 has previously been optimized by Liu et al. [19] (and unpublished data) where functionality of the ion channel was verified in a voltage sensitive dye (VSD) assay. Transfection was optimized for total amount of DNA and ratio of GABA_A_ (α2, β3, γ2) to YFP-H148Q/I152L plasmid. Results showed that 300 µg of total DNA containing 50% YFP-H148Q/I152L plasmid was optimal for functionality of GABA_A_ in CHO-K1 cells and generated the largest assay window in a GABA concentration-response experiment (data not shown). Cells were cryopreserved directly after electroporation and no change in assay performance was identified after at least 6 months storage in −150°C.

### I^−^ Affects Basal YFP Quench

**Figure 1:**
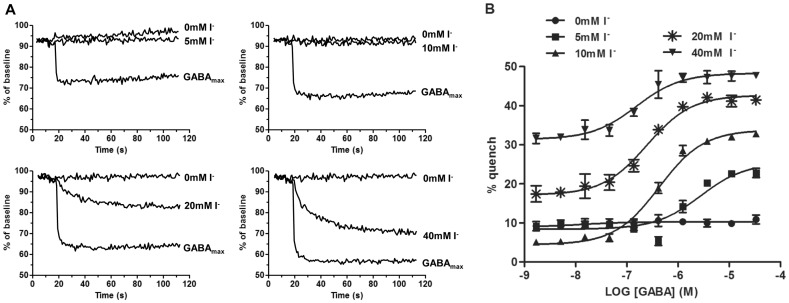
Basal I^−^ Permeability at Different I^−^ Concentrations. YFP-GABA_A2_ cells were exposed to different concentrations of I^−^ in the absence or presence of GABA. Data at 1–10 s was adjusted to a baseline of 100%. A) Representative time-courses of fluorescent quench showing basal I^−^ permeability and maximum GABA response (GABA_max_) of cells exposed to 5 mM, 10 mM, 20 mM and 40 mM NaI buffer. 0 mM NaI buffer is included in each graph as a reference. B) Concentration-responses of GABA with 0 mM, 5 mM, 10 mM, 20 mM and 40 mM NaI buffer showing I^−^ concentration-dependent baseline shift. The data represented are mean ± SD of quadruplicate wells. The experiment was repeated twice with similar results.

As a means of optimizing signal to background in the assay, we investigated buffers with different I^−^ concentrations (0 mM, 5 mM, 10 mM, 20 mM, 40 mM) by exposing cells to NaI buffer in the absence and presence of GABA (Figure 1). Figure 1A shows agonist-independent quench for the different I^−^ concentrations. Higher I^−^ concentrations, 20 mM and 40 mM, showed a significant agonist-independent YFP fluorescence quench, whereas lower concentrations, 5 mM and 10 mM, generated no, or only minimal, basal quench. Figure 1B demonstrates how the baselines and assay windows were affected when concentration-response curves of GABA were generated in 0, 5, 10, 20 and 40 mM NaI buffers. The increased basal quench observed at higher I^−^ concentrations, 20 mM and 40 mM, resulted in a baseline shift of the concentration-response curve and consequently reduced the assay window.

**Figure 2:**
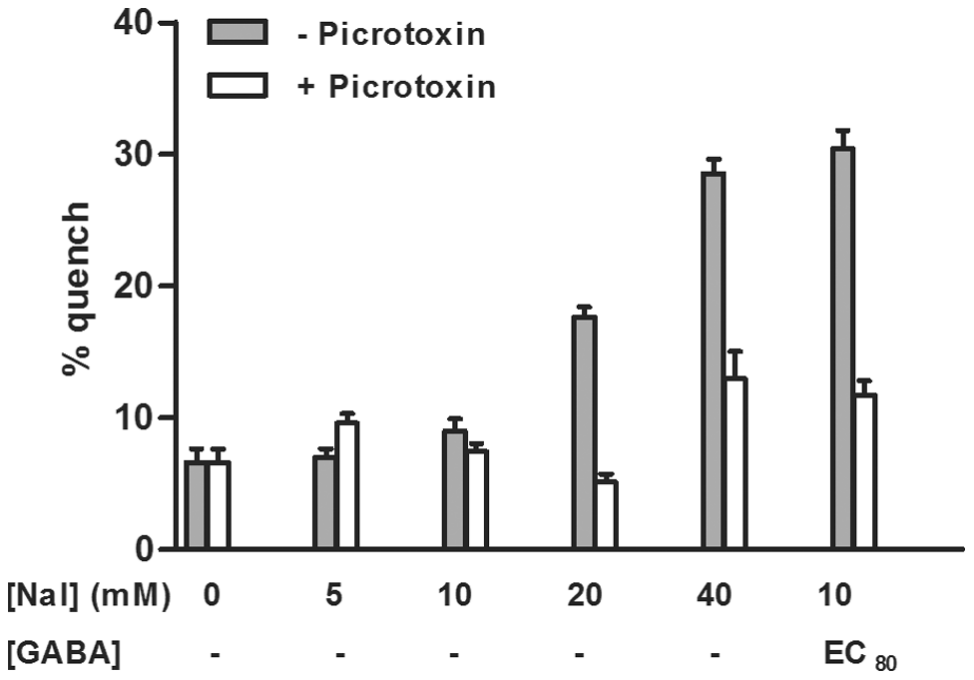
Blocking of GABA_A_ Ion Channel with Picrotoxin. YFP-GABA_A2_ cells were exposed to 100 µM Picrotoxin for 15 min prior to addition of different concentrations of I^−^. The bars furthest to the right represent activation with EC_80_ of GABA in 10 mM NaI buffer and blocking with 1 mM Picrotoxin. The graph shows representative mean data ± SD from two independent experiments.

To determine if the basal I^−^ permeability was GABA_A_ ion channel specific, 40 mM NaI buffer was added to CHO-K1 cells transfected with YFP-H148Q/I152L only. Basal quench was similar to that of buffer with 0 mM I^−^, hence no GABA_A_ independent quench could be detected (data not shown). To further verify that I^−^ was transported through the GABA_A_ ion channel 100 µM Picrotoxin, a non-competitive GABA_A_ antagonist, was added to YFP-GABA_A2_ cells 15 minutes prior to stimulation with buffer containing different I^−^ concentrations. Picrotoxin reduced basal quench to background levels (Figure 2). In addition, 1 mM Picrotoxin blocked YFP quench in cells activated with EC_80_ of GABA (Figure 2). For optimal assay performance a large assay window with minimal background quench is desired. Our results show that basal quench occurs at higher, but not lower, I^−^ concentration specifically through the GABA_A_ ion channel.

### I^−^ Influences GABA Potency

**Figure 3:**
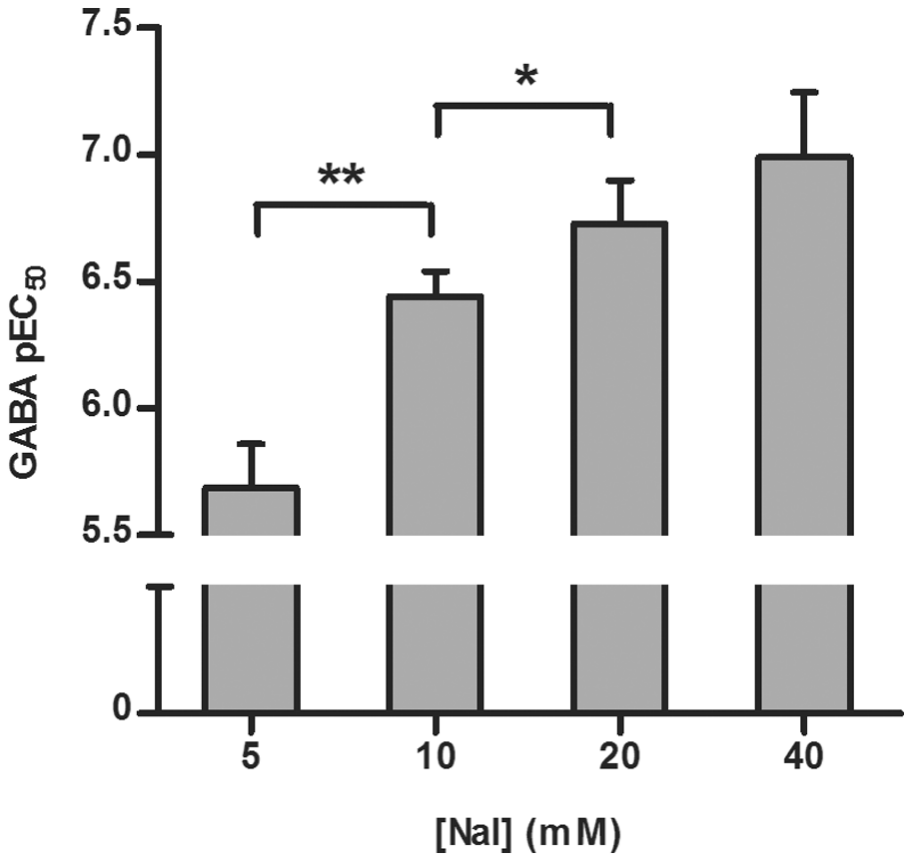
The Influence of I^−^ on GABA Potency. GABA pEC_50_ values for 5, 10, 20 and 40 mM I^−^ showing statistical significance at p<0.01 (**) and p<0.05 (*) when comparing pEC_50_ values at different I^−^ concentrations. The graph shows mean ± SD of two experiments.

The above results indicate that appropriate I^−^ concentration is important for optimal assay performance. To fully understand its implications, concentration-responses of GABA were determined and EC_50_ values calculated in the presence of different I^−^ concentrations. Analyzing the pEC_50_ values, a trend of increasing pEC_50_ with increasing I^−^ concentration was observed (Figure 3). Analysis of variance with two-sided tests was performed to determine the statistical significance of the pEC_50_ values for the different I^−^ concentrations. Differences in pEC_50_ turned out to be statistically significant at *p*<0.01 (**) when comparing 5 mM and 10 mM I^−^ and *p*<0.05 (*) when comparing 10 mM and 20 mM I^−^ (Figure 3). These results emphasize the importance of careful optimization of I^−^ concentration in the YFP assay. To reduce the artifact of agonist-independent I^−^ permeability while maintaining high signal to background and consistent data in GABA concentration-response mode, 10 mM I^−^ was considered to be the optimal concentration in assay.

### Antagonist Effect of Bicuculline

**Figure 4:**
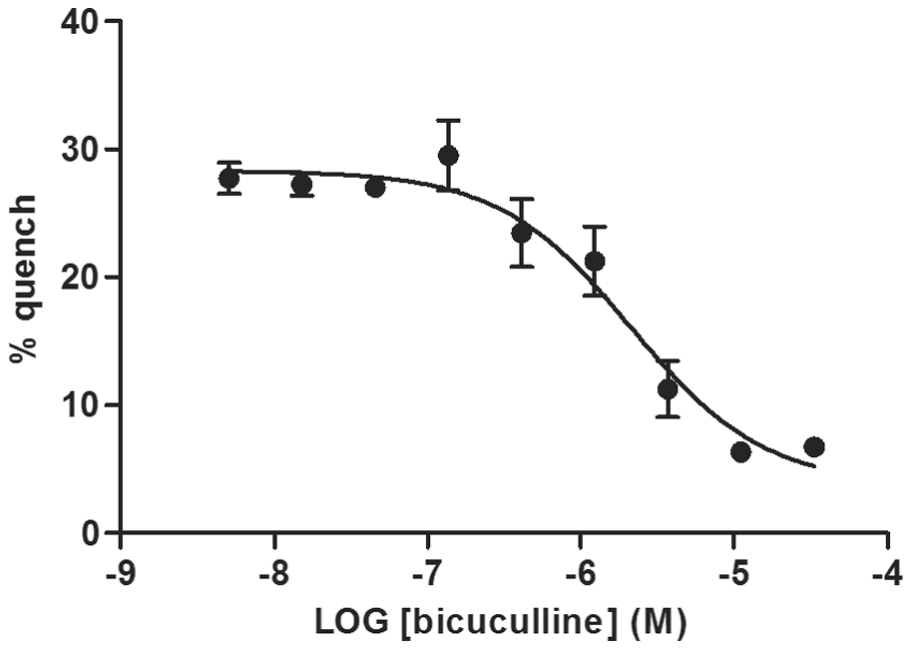
Inhibition of GABA_A_ with Bicuculline. Concentration-response of Bicuculline was added to YFP-GABA_A2_ cells 15 min prior to addition of EC_80_ of GABA in 10 mM NaI buffer. Data are mean ± SD of triplicate wells. IC_50_  = 2.14 µM.

To confirm the agonist mediated functionality of transiently transfected cells, a competitive GABA_A_ antagonist, Bicuculline, was used. Figure 4 shows Bicuculline inhibition of the ion channel when stimulated with EC_80_ of GABA. IC_50_ of Bicuculline was calculated to be 2.14 µM.

### Positive Allosteric Modulation of GABA Signal in YFP-GABA_A2_ Cells

**Figure 5:**
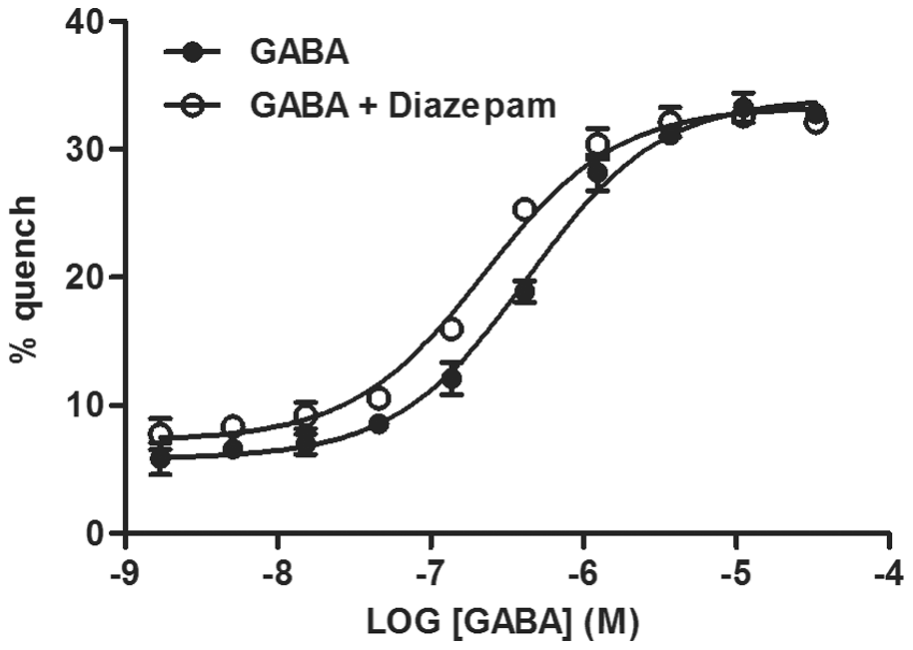
Modulation of GABA with Diazepam. YFP-GABA_A2_ cells were pre-incubated with DMSO or 1 µM Diazepam 15 minutes prior to addition of a concentration-responses of GABA at 10 mM I^−^. The graph shows representative mean data ± SD of quadruplicate wells. The experiment was repeated 3 times with similar results.

**Figure 6:**
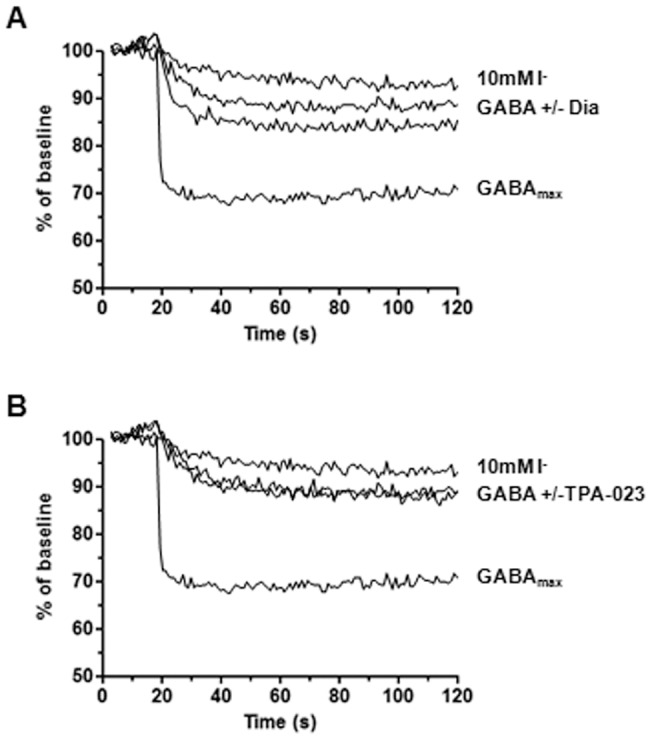
Modulation of GABA signal with Diazepam (Dia) and TPA-023. YFP-GABA_A2_ cells were exposed to EC_20_ of GABA alone or in the presence of 1 µM of modulator. Data at 1–10 s was adjusted to a baseline of 100%. Representative time-courses of fluorescent quench at basal (10 mM I^−^) and maximum GABA response (GABA_max_). A) Modulation of GABA EC_20_ quench showing a significant increase in the presence of Diazepam. B) No significant change of GABA EC_20_ quench in the presence of TPA-023. The experiment was repeated three times with similar results.

To investigate the YFP assay's suitability for screening of positive allosteric modulators, GABA concentration-response curves in the absence and presence of Diazepam, a strong positive allosteric modulator, were generated (Figure 5). Upon Diazepam addition the GABA EC_50_ decreased from 0.34 µM±0.08 µM (n = 3) to 0.20 µM±0.02 µM (n = 3) which equals a 1.7 fold EC_50_ shift. To further investigate the YFP assay's ability to distinguish GABA modulation, concentration-responses of GABA in the presence of 7 known modulators were determined. A pooled estimate of variability was used to calculate the standard error of the difference between two compounds' pEC_50_ values (average of 3 occasions). The data show that known modulators yielding a ≥1.40 times shift in GABA EC_50_, or a difference in pEC_50_ ≥0.15, compared to GABA, could be considered to have a statistically significant effect on GABA potency with 95% confidence, when averaging across 3 occasions (Table 1). Consequently, Diazepam, Lorazepam, Clobazam and Alpidem are modulators that have a significant positive allosteric effect on GABA (Table 1). However, we were not able to detect any significant allosteric modulation using the weak modulators TPA-023 or L-838417 (Table 1 and Figure 6).

**Table 1:** Modulation of GABA Signal with Allosteric Modulators.

Compound	pEC_50_	pEC_50_ difference	Significance 95% confidence
GABA	6.48±0.11	0	-
Diazepam	6.71±0.05	0.23	Significant difference
Lorazepam	6.71±0.09	0.23	Significant difference
Clobazam	6.66±0.05	0.18	Significant difference
Alpidem	6.84±0.07	0.36	Significant difference
L-838417	6.47±0.11	−0.01	non-significant
TPA-023	6.38±0.10	−0.10	non-significant
Desmethyl-clobazam	6.53±0.07	0.05	non-significant

Modulator pEC_50_ values were calculated from GABA concentration-response curves in the presence of 1 µM modulator. pEC_50_ differences were calculated from [Log (EC_50_ GABA alone) – Log (EC_50_ GABA with modulator)] and stated as statistically significant or non-significant at p<0.05. A pooled estimate of variability was used to calculate the standard error of the difference between two compounds' average of 3 occasions pEC_50_. The 95% confidence interval was calculated as 2× this standard error. Taking the anti-log converts this to a fold change (ratio) between two EC_50_ values.

## Discussion

In this study we establish a YFP assay capable of detecting GABA_A_ ion channel activity and modulation using transiently transfected YFP-GABA_A2_ cells. We show that transient transfection using MaxCyte STX electroporation instrument can be used to successfully generate functional YFP-GABA_A2_ cells. In addition, we show that the transfected cells can be cryopreserved, stored long term and subsequently thawed and used as assay-ready cells. The YFP assay proves to be fast and inexpensive with no need for dye loading, commercial kits or costly reagents. Previously, this assay has been used to study GABA_A_ functionality using GABA and the competitive GABA_A_ antagonist Bicuculline [15], [17]. We demonstrate for the first time how the YFP assay can be used to study agonist modulation. Studying the characteristics of the assay, we show that I^−^ concentration affects not only basal quench but also GABA potency, something that has not been reported before. Furthermore, it is a robust assay that can be used to distinguish small differences in modulator efficacy and has the potential to be used to discover novel modulators of the GABA_A_ ion channel in an HTS format.

To study ion channel functionality certain basic criteria of the cell line need to be considered. These include high transfection efficiency and low endogenous ionic current. CHO-K1 cells were chosen in this study since they are efficiently transfected, are highly viable after electroporation, show good transgene expression and have low expression of endogenous ion channels [20]. Transient transfection offers speed and flexibility which enables rapid assay evaluation and development compared to stable cell line generation. In addition it provides the possibility to titrate target expression. In the case of targets with multiple subunits, e.g. GABA_A_, it simplifies systematic titration of DNA amount to achieve optimal subunit composition. The difficulties associated with generation of functional stable GABA_A_ cell lines, the complexity of subunit compositions and the limited diversity of commercial cell lines makes transiently transfected cells an attractive alternative. Small-scale transfections to generate GABA_A_ cells using lipid-based reagents are common [15], [17], [19], although successful generation of transiently transfected GABA_A_ cells using large-scale electroporation has previously not been reported. This approach will provide a powerful tool when studying GABA_A_ ion channel pharmacology and diversity.

Genetically modified YFP-H148Q/I152L has the capacity to bind small anions resulting in quench of its fluorescence. The relative affinity of anions to YFP-H148Q/I152L are I^−^>NO_3_^−^>Br^−^>Cl^−^ [12]. This characteristic, along with its intracellular expression, photostability and fluorescent properties makes it suitable as an intracellular sensor to study anionic influx through ion channels. Since the reversal potential of Cl^−^ across the cell membrane is close to the resting potential of the cell, the driving force for Cl^−^ flux through the GABA_A_ ion channel is small. This low electrochemical driving force across the membrane as well as a fairly low affinity of Cl^−^ for YFP-H148Q/I152L has led to replacement of Cl^−^ with I^−^ for optimal assay performance when studying GABA_A_ in the YFP assay. In addition, I^−^ has been demonstrated to have a higher permeability than Cl^−^ through the GABA_A_ ion channel, with a permeability sequence of I^−^>Br^−^>Cl^−^>F^−^ [21]. Basal I^−^ permeability can occur through GABA_A_ ion channel or through other ion channels or transporters. This could be due to spontaneous opening of the ion channel with high extracellular I^−^ working as a driving force or due to I^−^ acting as a GABA_A_ modulator [21]. Basal I^−^ permeability results in quenching of YFP signal and reduces the maximum relative quench that can be achieved upon agonist activation of the ion channel. Previously, GABA_A_ ion channel functionality has not been studied in CHO-K1 cells using the YFP assay. To optimize signal to background different I^−^ concentrations, ranging from 5–40 mM final concentration, were investigated and 10 mM was determined to be optimal in terms of minimal basal quench, maximum assay window and data consistency. Previously described YFP assays used a range of I^−^ from 72.5–120 mM I^−^. These reports describe different Cl^−^ channels, CFTR and glycine, and different cell backgrounds including; Human embryonic kidney (HEK293); Fisher rat thyroid; 3T3 and CHO-K1 cells [11], [13], [15]. Together these data show that optimal I^−^ concentration in the YFP assay is dependent on the cell background and on the characteristics of the ion channel used. Furthermore, the influence of I^−^ concentration on basal quench, as well as our data showing decrease in GABA potency with increasing I^−^ concentration, demonstrates the importance of careful optimization to identify the most suitable assay concentration. Robertson describes an increase in negative current at negative potentials when performing single-electrode voltage clamp of GABA stimulated dorsal root ganglion neurons in the presence of I^−^ compared to Cl^−^ [21]. He hypothesizes that I^−^ prolongs the opening time of the ion channel possibly by interacting and modulating the kinetics, thereby increasing the time that GABA binds to the ion channel. Based on this, it is possible that the modulating effect of I^−^ is concentration dependent and as a consequence affects GABA potency, as observed in our assay. Furthermore, control experiments using Picrotoxin, a non-competitive GABA_A_ antagonist, supported the theory that I^−^ influx occurs selectively through the GABA_A_ ion channel.

Differences in GABA potency have been reported depending on GABA_A_ ion channel subunit composition, assay and cell type used. There are only few reports in the literature stating GABA EC_50_ values using a α2β3γ2 subunit composition. It has been shown that GABA sensitivity is increased for αβ composition compared to αβγ [3], [17]. Using transiently transfected YFP-GABA_A2_ cells in the YFP assay we obtained a GABA EC_50_ of 0.34 µM±0.08 µM. Lui et al. obtained a GABA EC_50_ of 2.2 µM with HEK 293 cells expressing the α2β3γ2 ion channel subtype in a membrane potential assay [19]. This six fold difference in GABA EC_50_ may be accounted for by the different cell backgrounds used, HEK293 and CHO-K1. In addition, the membrane potential assay measures outward current of ions whereas the YFP assay measures influx, which is the physiological current of ions upon GABA stimulation. To conclude, the aim with the YFP assay is not to obtain absolute potency measurements, but to measure the modulation of GABA activation of the channel in order to compare different modulators.

DNA ratio used for transfection does not necessarily correlate with protein subunit expression. Functional and pharmacological experiments are therefore needed to ensure correct subunit assembly and ion channel function [3], [22]. The subunit composition determines the GABA sensitivity and the pharmacological properties of the channel. Experiments were performed on cells expressing GABA_A_ α2β3γ2 optimized to yield a composition of two α2, two β3 and one γ2 subunit stoichiometry. Functionality of the GABA_A_ ion channel was confirmed by agonist activation using GABA as well as inhibition using Bicuculline and Picrotoxin. The benzodiazepine site is located at the interface of the α and γ subunits and a correct subunit composition of the channel is required to yield agonist modulatory effect with a benzodiazepine-class compound. We report modulation of GABA activation using different positive allosteric modulators. The strong positive modulator Diazepam shifted GABA EC_50_ 1.7 fold, which corresponds to a pEC_50_ difference of 0.23, compared to GABA alone. This may be considered a small shift and can be compared to a pEC_50_ difference of 0.41 generated with VSD assay by Liu and coworkers [19]. However, when testing 7 known modulators, statistical analysis showed that our YFP assay can distinguish pEC_50_ differences as small as 0.15 when averaging across 3 occasions. A direct comparison of EC_50_ values of these 7 different modulators compared to literature values is however not relevant, given the difference in modulator effect observed using different subunit compositions of the GABA channel and the variation in EC_50_ values reported from different cell backgrounds and assays. The ability to modulate GABA potency upon modulator addition confirms that our YFP-GABA_A2_ cells express functional GABA_A_ α2β3γ2 ion channels with correct subunit composition containing a benzodiazepine binding site. It further implies that the assay can be used to screen for modulators and rank order modulators with small pEC_50_ differences. However, using the current assay protocol, we were not able to detect a statistically significant effect of known weak positive allosteric modulators. In mouse fibroblast L(tk^−^) cells expressing GABA α2β3γ2, relative in vitro efficacy of TPA-023 or L-838417 was reported to be 11% and 34% respectively, measured as the potentiation of GABA EC_20_ compared to a 100% response from a classical benzodiazepine full agonist, chlordiazepoxide [23], [24]. The inability to measure modulation of the GABA signal with these compounds is a limitation with the YFP assay compared to the VSD assay. Using the VSD assay Liu and coworkers detected modulation of GABA signal using the weak modulator L-838417 [19]. A way to improve the sensitivity of the YFP assay may be to further optimize the I^−^ concentration. Using different I^−^ concentrations we observed a trend of increased positive modulator efficacy with decreased I^−^ concentrations, (data not shown). However, a consequence of decreased I^−^ concentration is a decreased assay window and an increased variability of the assay. Taken together, increasing the sensitivity of the YFP assay by decreasing the I^−^ concentration results in reduced assay reproducibility and robustness. Even though there is an obvious challenge of using the YFP assay for screening of positive GABA_A_ modulators in an HTS format, our initial results indicate that the assay produces robust data for moderate to strong modulators.

Although it is beyond the scope of the current study, screening a larger set of compounds would confirm the YFP assay's robustness and provide an answer about the utility of the assay in HTS to identify positive allosteric modulators of GABA_A_. To find subtype selective modulators of GABA_A_, cells with different subunit compositions would need to be generated and used in screening. Our work demonstrates how to successfully generate YFP-GABA_A2_ cells. To generate transiently transfected cells expressing GABA_A_ with a different set of subunits, DNA ratio optimization could be evaluated with functional characterization similar to what has been described here. In conclusion, using the YFP assay with transiently transfected CHO-K1 cells provides a flexible means for discovering novel GABA_A_ modulators.
